# A Novel Method for Improved Network Traffic Prediction Using Enhanced Deep Reinforcement Learning Algorithm

**DOI:** 10.3390/s22135006

**Published:** 2022-07-02

**Authors:** Nagaiah Mohanan Balamurugan, Malaiyalathan Adimoolam, Mohammed H. Alsharif, Peerapong Uthansakul

**Affiliations:** 1 Department of Computer Science and Engineering, Sri Venkateswara College of Engineering, Sriperumbudur 602117, India; 2 Department of Computer Science and Engineering, Saveetha School of Engineering, Saveetha Institute of Medical and Technical Sciences, Thandalam 602105, India; m.adimoolam@gmail.com; 3 Department of Electrical Engineering, College of Electronics and Information Engineering, Sejong University, Seoul 05006, Korea; malsharif@sejong.ac.kr; 4 School of Telecommunication Engineering, Suranaree University of Technology, Nakhon Ratchasima 30000, Thailand

**Keywords:** machine learning, deep learning, network traffic, traffic prediction, reinforcement learning, internet traffic

## Abstract

Network data traffic is increasing with expanded networks for various applications, with text, image, audio, and video for inevitable needs. Network traffic pattern identification and analysis of traffic of data content are essential for different needs and different scenarios. Many approaches have been followed, both before and after the introduction of machine and deep learning algorithms as intelligence computation. The network traffic analysis is the process of incarcerating traffic of a network and observing it deeply to predict what the manifestation in traffic of the network is. To enhance the quality of service (QoS) of a network, it is important to estimate the network traffic and analyze its accuracy and precision, as well as the false positive and negative rates, with suitable algorithms. This proposed work is coining a new method using an enhanced deep reinforcement learning (EDRL) algorithm to improve network traffic analysis and prediction. The importance of this proposed work is to contribute towards intelligence-based network traffic prediction and solve network management issues. An experiment was carried out to check the accuracy and precision, as well as the false positive and negative parameters with EDRL. Also, convolutional neural network (CNN) machines and deep learning algorithms have been used to predict the different types of network traffic, which are labeled text-based, video-based, and unencrypted and encrypted data traffic. The EDRL algorithm has outperformed with mean Accuracy (97.20%), mean Precision (97.343%), mean false positive (2.657%) and mean false negative (2.527%) than the CNN algorithm.

## 1. Introduction

Internet data traffic has been enormously burst out, due to the introduction of big data capable data, along with the invention of speed network components. The resources of such a network are essential, and it have to be utilized for the intended purpose; additionally, it is a very challenging task to monitor and predict data traffic for various reasons. For data traffic prediction, manual processing-based prediction and artificial intelligence-based methods and techniques have already been deployed. Some of the methods and techniques are as follows. (i) The prediction of daily internet traffic using a data mining technique for smart university application [1]; (ii) A low complexity-based boost machine learning algorithm with classification and regression to predict internet data traffic from weak learning to strong learning [2]; (iii) A double exponential predictor [3], based on artificial neural network (ANN), classic time series, and wavelet transform-based predictors; (iv) A deep learning-based prediction [4] for metropolitan area network traffic; (v) A neural network ensemble [5] for internet traffic forecasting.

Network traffic prediction [6] is inevitable, due to the cost of bandwidth, time complexity measurement, data prediction, suspicious traffic identification, and so on. The traffic of network impact is directly proportional to the bandwidth and life span of network and multi-user identification. A recurrent neural network (RNN) was adopted to find network traffic for proactive network management and planning [7]. The RNN prediction formula is as shown in Equation (1).

(1) $$y_{i}=w_{i}h_{i}+y_{i}-1$$

where *y_i_* is the predicted value at time *i*, *w_i_* is weight of input, *h_i_* is hidden layer state at time *i*, and *y_i_*_−1_ is predicted value at time *i* − 1 [8]. The GEANT backbone network was tested with the network structure of RNN and optical network parameters with 200 epochs. Yet another work was discussed regarding four different algorithms for predicting network traffic [9]. Those algorithms were RNN, deep learning stacked auto-encoder, multilayer perceptron (MLP), and MLP with back-propagation. Aspects such as adaptive application, bandwidth detection, congestion control, and anomaly detection and admission control network traffic and have been discussed with time series internet traffic prediction.

The application of network traffic analysis and prediction consist of bandwidth monitoring, data analysis, efficient network management for intended users, and so on. Little research has been carried out to measure the bandwidth efficiency. Work regarding bandwidth utilization and forecasting model was discussed for bandwidth utilization with ARIMA and SNMP setups [10]. The computational time was measured, and it was achieved at 83.2%, along with forecast error and standard deviation.

Yet another application-oriented network traffic prediction was performed for measuring the accuracy and timely internet traffic information [4]. In this work, the proposed mechanism has detected regarding network traffic for anomaly detection, admission control, bandwidth allocation, and congestion control with big traffic data and deep architecture model-based internet traffic flow prediction. The novelty has been achieved with special and temporal correlations, as well as the glow data character approach. The training data set was trained in the greedy layer-wise fashion. The dataset is taken from China Unicom for network work utilization. Yet another work surveyed real world network traffic prediction with various machine learning algorithms with a cognitive approach [11]. Here, the applications are coined based on their classifications for threat category, regression for value prediction, and ranking for ordering traffic. The learning algorithms discussed here were neural network, linear time series models, principal component analysis (PCA), linear regression (LR), statistical model, and support vector machine (SVM) for either long-term or short-term predictions. The application’s performance measures have been taken as data availability and system complexity for both local and wide-area networks. From discussed techniques, the applications supported were cellular traffic, optical networks, LTEs network, IP networks, TCP traffic, MPEG, JPEG traffic, Ethernet traffic, and many more.

## 2. Related Works

### The Applications of Network Traffic Analysis

The major applications of network traffic prediction are network management, resource allocation, quality of service (QoS) from the internet service provider (ISP), cyberspace security protections, and malware detection [12]. The ISCX and QUIC public dataset was used here to measure the performance of traffic, with a proposed method called multi-task learning framework. Yet another work was related to the online application of current internet performance measures, as determined by analyzing encrypted packet, virtual private network, and non-VPN traffic using the proposed method, referred to as deep packet. This work had taken file transfer protocol (FTP) and peer-to-peer (P2P) network traffic. The recall performance was measured for the UNB ISCX VPN and non-VPN dataset [13]. The network classifier approach was deployed in hyper-text transfer protocol (HTTP) and session initiation protocol (SIP) [14]. Performance measures such as duration, latency, and traffic volume were measured using RNN and CNN learning algorithms.

In the past decade, more than 40 research works have been introduced that discussed network traffic analysis with manual traffic prediction, machine learning-based network traffic prediction, and deep learning-based traffic prediction. Most of the work used machine and deep learning algorithms to predict network traffic. A work was introduced to predict network traffic using a time series approach with recurrent neural network (RNN) [7]. Also, the variation of RNNs were analyzed using past network traffic dataset. Its performance was measured using the GEANT research and educational network. An experiment was conducted with 200 epochs with a learning rate of 0.01 to 0.5. The performance of variant long short-term memory (LSTM) was better than other variants of RNN.

For network traffic, most cited articles were related to deep learning and machine learning traffic identification algorithms. One work coined a suitable lightweight framework with a deep learning algorithm. These frameworks have penetrated the encrypted traffic, classified the deep full range, and detected the intrusion with two datasets [15]. Yet another application, i.e., the user activity monitoring-based network traffic, was developed using the machine learning algorithm [16]. K-mean and random forest (RF) algorithms have been used to measure the network traffic QoS, accuracy, and real time traffic generated with time bound. A network management-based traffic classification with software defined network (SDN) was tested with a CNN and stacked auto encoder (SAE). This proposed work was used for online traffic service. Recall, accuracy, and precision were measured with a deep learning algorithm [17]. Finally, Table 1 depicts the methods and classes of machine and deep learning algorithms. The CNN prediction formula is given in Equation (2), and it is a neuron calculation for traffic.

(2) $$y_{i}=b_{i}+\sum_{i=1}^{n}w_{i}\times x_{i}$$

where *y_i_* is the neuron calculation, *w_i_* is the weight matrix of input, *x_i_* is input, and *b_i_* is the bias of the neuron.

Even though a considerable amount of research has been carried out, accurate predictions and huge traffic predictions remain unclear. So, it is important to carry out inevitable solutions to predict traffic from huge datasets. On the other hand, using CNN for traffic predictions has to be checked, in regard to whether there is less energy consumption and simple infrastructure-based mechanism for traffic prediction of the internet world. Thus, the enhanced reinforcement learning algorithm would be the best to choose. It is important to sort out the aim proposed in this work. The aim of this work is to predict network traffic and measure its related performance measures, such as accuracy and precision, as well as false positive and negative rates, using the EDRL algorithm and comparing its performance with the KNN and CNN algorithms.

## 3. Materials and Methods

This research work was carried out in the machine learning lab, Saveetha school of Engineering, Saveetha University. The proposed algorithm, i.e., the EDRL algorithm, was compared with CNN. Group 1 was taken as the proposed EDRL and CNN was taken as group 2. For each group, 25 iterative samples are set for experiment, with a dataset size of 54,000. A total of 80% of the pre-test power (G power) was obtained, along with alpha value 0.05. The dataset was collected from ISCXVPN2016 [18] for virtual private network (VPN) and non-VPN. The size of the dataset is almost 15 GB of ARFF file format, with a set of attributes for instance sharing. The dataset is traced with scenarios with different traffics from networks. The dataset is divided into two partitions for testing and training purposes. The training set contains 80%, and the testing set contains 20%.

To understand how EDRL is better than reinforcement learning (RL), deep reinforcement learning (DRL), and deep learning (DL), the following (Table 2) illustrates the relationship between the input and methods, along with the policies and problems for different machine and deep learning algorithms.

### 3.1. The EDRL Algorithm

EDRL processes input with DL networks to grain the output of accurate network traffic; then, the gained output undergoes reinforcement learning with policy optimization using policy gradient methods, thus achieving an EDRL method with higher accuracy than normal. This method entirely undergoes a multi-layer perceptron neural network for function approximation and various reward functions. The mechanism works after DNN checks and pre-processes the network traffic dataset, using Monte Carlo learning check flow independently and dictionary-based learning to reiterate the RL, along with reward mechanism, achieves fine-tuned accuracy.

### 3.2. Dataset Pre-Processing

Data pre-processing is the way to process the data for training and testing. Let *X* be the set of data taken from the dataset and Equation (3), which is called to pre-process the network traffic data for prediction with accuracy and precision.

(3) $$x=\frac{X-X_{min}}{X_{max}-X_{min}}$$

### 3.3. Feature Engineering

To enhance the accuracy, various features are selected and form a feature set, A = a_1_ to a_n_. These features are based on the parameters of network traffic and types of applications of network traffic. Features such as average segment size, window size, round trip delay time (RTT), variance of packet, actual data size, client port number, and server port numbers were taken to predict network traffic; the features are represented as F in Equation (4), and the description is illustrated in Table 3.

(4) $$F=\left\{f_{1},{\;f}_{2},f_{3}\ldots f_{7}\right\}$$

The classes as www packets, P2P packets, mail packets, database packets, and multimedia packets and its classes are represented as C in Equation (5), and its notation and applications are listed in Table 4. The classes of network c_1_ to c_5_ of network traffic are processed with EDRL, with deep penetration of functions f_1_ to f_7_, as listed in Table 3. This function can trace the format of network traffic data, such as general browsing data, torrent streaming, SMTP, POP, MIME, IMAP, SQL net, and video storage server of YouTube. The function f_3_ is checking the variance of packets to check traffic very accurately. RTT checks the delay time occurrence of network traffic. Further, the average size of the segment elaborates on how the network traffic changes from time to time.

(5) $$C=\left\{c_{1},c_{2},c_{3}\ldots c_{5}\right\}$$

### 3.4. The DNN Multi-Layer Perceptron Method

**Figure 1:**
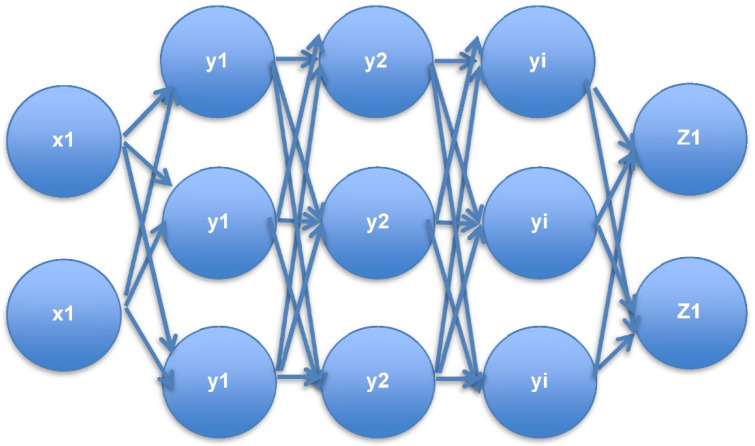
DNN multilayer perceptron for network traffic processing.

It is a sophisticated model with a mathematical-based complex data processing network. It is the technology built-based model for simulating a neural connection. This is here to represent the recognition of pattern of network traffic dataset by passing input, and it will process through the neural connection with various layers, as shown in Figure 1. Usually, DNN has an input layer and output layer, which are placed between many hidden layers. After the input layer accepts the input as traffic network data, further hidden layers perform the sorting and ordering of data, and finally outputting the layer for getting pre-processed data of traffic network. The main functionality of this DNN is that it handles the unlabelled or unstructured data and introduces multilayer perceptron. Further, it is clear that this input layer has to feed the data of network traffic, both encrypted and unencrypted, including the different functions of f_1_ to f_7_ and different classes of c_1_ to c_5_. Then, the neighbor of this input layer trains the dataset and finds its threshold values, until deep EDRL getts its corresponding traffic classification clearly. This can be achieved with the help of the output layer of DNN.

### 3.5. Monte Carlo Learning for Network Traffic Analysis

Monte Carlo learning (MCL) is a Q-learning (QL) method of reinforcement learning (RL), which calculates the policy with agents to do research to find which policy gains more rewards. For traffic to be predicted accurately, reward functions play an inevitable role. Additionally, for the types of network traffic, relative rewards were calculated. For network traffic analysis, the Markov decision process (MDP) was used, and its components are A, P, R, RF, and S [19], where S is set of states in traffic analysis, A is set of actions in traffic analysis, R is reward for traffic analysis from one state to another state, P is set of policy, and, finally, RF is the reduction factor. The working mechanism of Q_L_ is that, for traffic analysis, there is a transition of state s_1_ (s_1_ ∈ S) to s_2_ (s_2_ ∈ S) for carryout action process (a ∈ A) with policy P (s_1_ to s_2_, a_1_), along with reward R (s_1_ to s_2_, a_1_), which could be calculated. This reward calculation will be introducing novelty for traffic prediction with the network dataset. This QL process is illustrated as Equation (6), as follows.

(6) $$Q_{L}\left(s_{i},a_{i}\right)={\;R}_{F}(\max(Q\left(s_{i}+1,a_{i}+1\right)+\;R\left(s_{i},a_{i}\right)$$

where (a_i_ ∈ A) is action set and this QL is the process to achieve maximum network traffic prediction, and this will be an iterative process to achieve the maximum reward with the sum of the reward, and it is expressed as Equation (7), as follows.

(7) $$Q\left(s_{i},a_{i}\right)=\left(\Delta -1\right)Q\left(s_{i}+1,a_{i}+1\right)+\;\Delta (R\left(s_{i},a_{i}\right)+R_{F}(\max\left(Q\left(s_{i}+1,a_{i}+1\right)\right)$$

Based on Equations (6) and (7) Monte Carlo QL is used to train the network traffic dataset as Equitation (8).

(8) $${X\;}_{t}={\;Q}_{L}\left(X_{\mathrm{low}},X_{\mathrm{high}}\right)$$

For network traffic prediction, the QL-based traffic prediction is coined as Algorithm 1, after calling EDRL.
**Algorithm 1:** Monte Carlo Learning for Network Traffic AnalysisPre-requisite: Pre-processed dataset (X) for number of iterations Assure: Max (R(X, A_i_) X = (X−X_min_)/(X_max_−X_min_) QL = X_max_ − X_min_*X_max+1_ − X_min+1_ R_i_(S_i_, S_i+1_) = max(1, R_i_(S_i_, S_i+1_)) F = {f_1_, f_2_, f_3_…f_7_} C = {c_1_, c_2_, c_3_…c_5_} For each S_i_ ∈ X do Q(s_i_,a_i_) = RF(max(Q(s_i+1_, a_i+1_) + R(s_i_, a_i_) Q(s_i_,a_i_) = (Δ − 1)Q(s_i+1_, a_i+1_) + Δ(R(s_i_, a_i_)+RF(max(Q(s_i+1_, a_i+1_)) R_i+1_(S_i_, S_i+1_) = R_i+1_(S_i_, S_i+1_) +1 End for Max (R(X,A_i_)

### 3.6. Agent—EDRL Traffic Model

**Figure 2:**
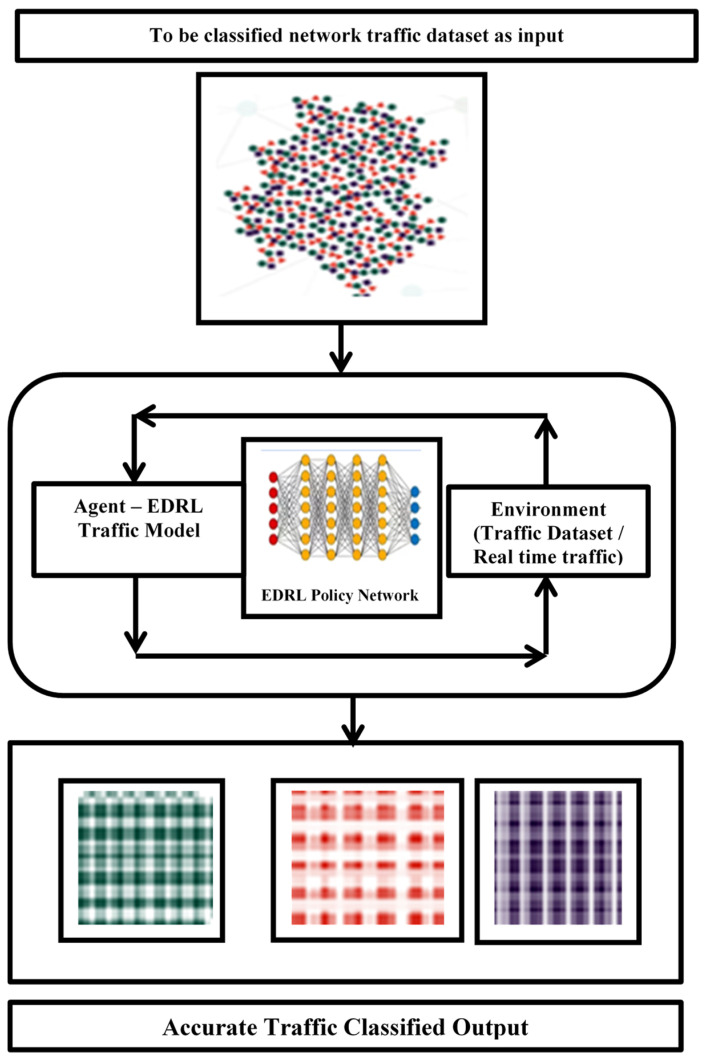
An EDRL architecture for network traffic analysis.

EDRL is one of the artificial learning algorithms that computes optimized output, thus enhancing its feedback, along with the deep learning concept. Pre-processed input collected from a dataset with non-encrypted and different application-based network traffics [18] is fed in EDRL algorithm. The dataset is trained in EDRL algorithm and predicts the data as classified 1 to N, and its accuracy has been measured as a parameter with an event-based approach, instead of time series; similarly, the experiment has been carried out with precision and false positive and negative rates. The EDRL architecture is shown in Figure 2. In this work, the network traffic of the encrypted and non-encrypted dataset is used. It consists of VPN- and non-VPN-based patterns. The encrypted dataset of the ARFF file format is essential for tracing network traffic. This ARFF also includes Skype and multimedia network traffic. This work has also traced the network traffic of www packets, P2P packets, mail packets, database packets, and multimedia packets.

This overall EDRL algorithm is coined as Algorithm 2 for network traffic prediction and accuracy measurement.
**Algorithm 2:** EDRL Algorithm for Network Traffic PredictionPre-requisite: pre-processed network traffic data from dataset Assure: max (precision), max (accuracy), min (falsepositive), min (falsenegative) QL = X_max_ − X_min_*X_max+1_ − X_min+1_ Call feature engineering function Call DNN multilayer perceptron method Call Monte Carlo learning for network traffic analysis Algorithm 1

### 3.7. Accuracy and Precision for Network Traffic Analysis

The accuracy of identifying network traffic rests on the closeness of the specific value, while the precision is the measurement of the closeness of the network traffic to each other while checking for network traffic prediction. Equation (9) represents the accuracy for the network traffic data.

(9) $$\mathrm{Accuracy}=\frac{\mathrm{TP}+\mathrm{TN}}{\mathrm{TP}+\mathrm{TN}+\mathrm{FP}+\mathrm{FN}}$$

where TP is true positive and TN is true negative, FP is false positive and FN is false negative. Equation (10) is a formula that is used to measure the precision of network traffic prediction.

(10) $$\mathrm{Precision}=\frac{\mathrm{TP}}{\mathrm{TP}+\mathrm{FP}}$$

### 3.8. Statistical Analysis

The measured traffic categories’, i.e., accuracy, precision, falsepositive and falsenegative, parameter performance values were collected by repeated experiments for 10 iterations, and these values were recorded. Further, the recorded values of each parameter were tabulated, and comparative graphs were drawn with help of a statistical IBM SPSS tool. The dependent and independent variables are represented to measure the best accuracy-incurring algorithm by comparing EDRL algorithm with CNN and KNN algorithm. Here, the feature of network traffic and classes act as a dependent variables and EDRL and CNN algorithms act as an independent variables; they are used to carry out the experiment to predict precise and accurate network traffic along with the falsepositive and falsenegative parameters.

## 4. Numerical Results

### 4.1. Accuracy Comparison

The implemented experiment results have been taken iteration-wise, and the group statistics were carried out with the IBM SPSS tool. For the 10 iterated samples of trained and tested datasets, an independent sample *t*-test was built. The Group 1 algorithm was taken as EDRL, and the group 2 algorithm was taken as the CNN algorithm. The statistical results’ were observed. Table 5 shows the group statistics of the EDRL and KNN algorithms, as well as EDRL and CNN. The experiment was carried out with 10 iterations of EDRL and compared with the CNN algorithms separately. It is noticed that mean accuracy of EDRL was 97.20%, and the CNN mean accuracy was 93.055%. The standard deviation is comparatively less 1.702% in the EDRL and CNN algorithms (2.298%). Further, the standard error mean was also 0.538% for EDRL, which is comparatively less than the CNN algorithm (0.7268%). The experiment inferred that the validation and trained accuracy increased when the number of iterations was increased for EDRL than for the KNN and CNN algorithms.

Further, the significance value was calculated between the EDRL and CNN algorithms using the SPSS tool comparing the independent *t*-test as analytics. Table 6 shows the significance value. The inference is that there is a significant difference between EDRL and CNN of 0.306. The inference further claims that there is slight difference between alpha test *p* = 0.05 with inferred difference.

### 4.2. Precision Comparison

Table 7 shows the group statistics of the EDRL and CNN algorithms’ precision for 10 iterations of EDRL and compared this with the CNN algorithms separately. It is noticed that mean precision of EDRL was 97.373%, whereas the CNN mean precision was 93.972%. The standard deviation was comparatively less, at 1.5189%, in the EDRL algorithm than the CNN algorithm (2.403%). Further, the standard error mean was 0.4803% for EDRL, which is comparatively less than the CNN algorithm (0.7594%). The inference is that the validation and trained accuracy increased when the number of iterations increased for EDRL, rather than the CNN algorithm.

The significance value was calculated between the EDRL, and the CNN algorithms’ precision, using the SPSS tool, compared the independent *t*-test as analytics. Table 8 is shows the significance value of the mean precision. The inference is that there is a significant difference between EDRL and CNN of 0.143. The inference further claims that there is slight difference between alpha test *p* = 0.05, with inferred difference.

### 4.3. False Positive Comparison

Table 9 shows the group statistics of the EDRL and CNN algorithms’ false positive rates for 10 iterations of EDRL, and they compared with CNN algorithm separately; we noticed that the mean false positive of EDRL was 2.657%, whereas the CNN mean false positive was 6.325%. The standard deviation is comparatively less 1.853% in the EDRL algorithm than the CNN algorithm (2.191%). Further, the standard error mean was 0.581% for EDRL, which was comparatively less than the KNN (0.731%) and CNN (0.693%) algorithms. The inference that the validation and trained accuracy increased when the number of iterations increased for EDRL, rather than the KNN and CNN algorithms.

The significance value calculated between EDRL and CNN algorithms’ false positive using SPSS tool using comparing independent *t*-test as analytics. Table 10 is showing the significance value of mean false positive. The inference is that there is a significant difference between EDRL and CNN as 0.143. The inference further claims that there is slight difference between alpha test *p* = 0.05 with inferred difference.

### 4.4. False Negative Comparison

Table 11 shows the group statistics of EDRL and CNN algorithms’ false negative. For 10 iterations of EDRL and compared with CNN algorithms separately and noticed that mean False negative of EDRL is 2.527% whereas CNN mean False negative is 5.675%. Standard deviation is comparatively less 1.227% in EDRL algorithm than CNN algorithm (1.992%). Further standard error mean is 0.381% for EDRL which is comparatively less than CNN algorithm 0.61643%. Inference that the validation and train Accuracy increases when number of iteration is increases for EDRL than CNN algorithms.

The significance value calculated between EDRL and CNN algorithms’ false negative using SPSS tool using the comparing independent *t*-test as analytics. Table 12 shows the significance value of the mean false negative. The inference is that there is a significant difference between the ERDL and CNN of 0.143. The inference further claims that there is a slight difference between the alpha test *p* = 0.05, with inferred difference.

### 4.5. Accuracy Comparison for EDRL and CNN Algorithms

**Figure 3:**
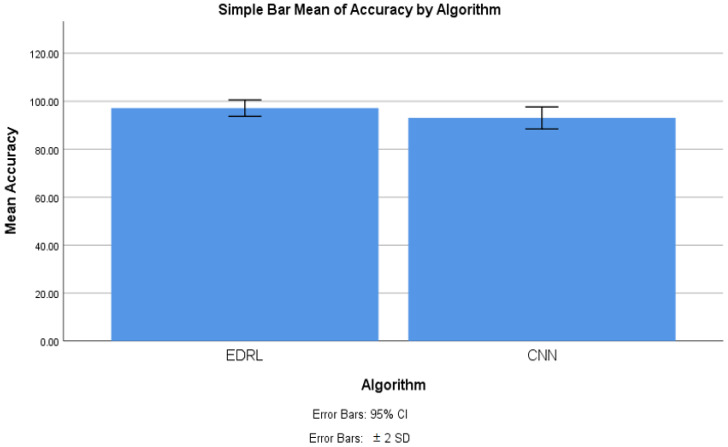
Mean accuracy comparison of EDRL and CNN, including error rates.

With the SPSS tool, the mean accuracy of the ERDL and CNN algorithms has been compared, as shown in Figure 3. The graph was generated with SPSS graph builder, with the X axis as the ERDL vs. CNN algorithms and Y axis as the mean accuracy. The standard deviation is set at ±2 with confidence interval of 95%. The mean accuracy of the ERDL algorithm was higher than the CNN algorithm. For the 10 epochs, the mean accuracy was considerably higher for the ERDL than the CNN algorithm, as the sample size increased and error rates decreased with the increasing sample size for two algorithms.

### 4.6. Precision Comparison for EDRL and CNN Algorithms

**Figure 4:**
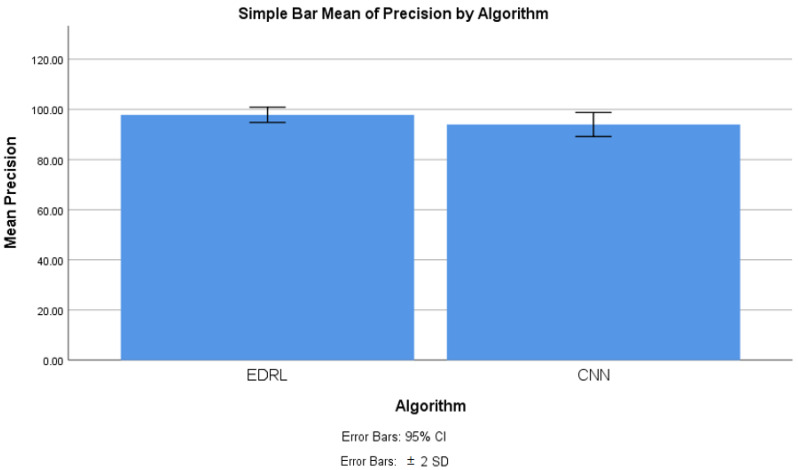
Precision comparison of EDRL and CNN algorithms with the error mean measure.

With the SPSS tool, the mean precision of the ERDL and CNN algorithms was compared, as shown in Figure 4. The graph was generated with the SPSS graph builder, with the X axis as the ERDL vs. CNN algorithms and Y axis as mean the precision. The standard deviation was set at ±2, and the confidence interval was 95%. The mean precision of the ERDL algorithm was higher than the CNN algorithm. For the 10 epochs, the mean precision was considerably higher for ERDL than the CNN algorithm, as the sample size increased and error rates also decreased, with increasing sample sizes for the two algorithms.

### 4.7. False Positive Comparison for EDRL and CNN Algorithms

**Figure 5:**
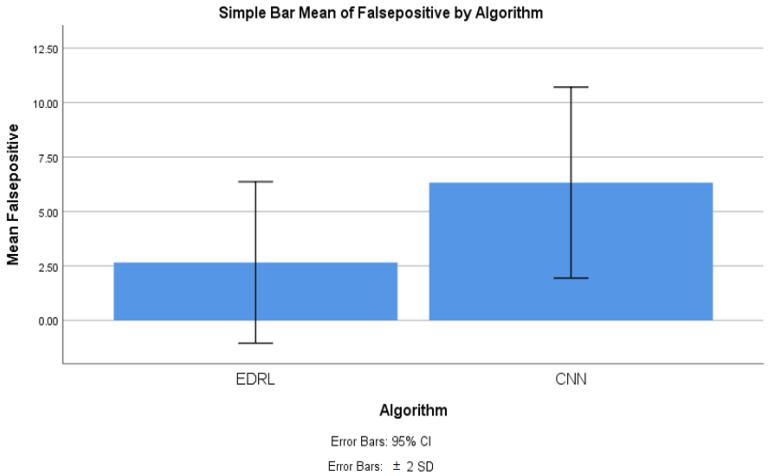
The false positive comparison between the EDRL and CNN algorithms with stand error bars with SD and confidence interval.

With the SPSS tool, the mean false positive of ERDL and CNN algorithms were compared, as shown in Figure 5. The graph was generated with the SPSS graph builder with the X axis as the ERDL vs. CNN algorithms and the Y axis as mean false positive. The standard deviation was set at ±2, and the confidence interval was 95%. The mean precision of the ERDL algorithm was higher than the CNN algorithm. For the 10 epochs, the mean false positive was considerably higher for ERDL than the CNN algorithm, as sample size increased and error rates decreased, with increasing sample sizes for the two algorithms.

### 4.8. False Negative Comparison for EDRL and CNN Algorithms

**Figure 6:**
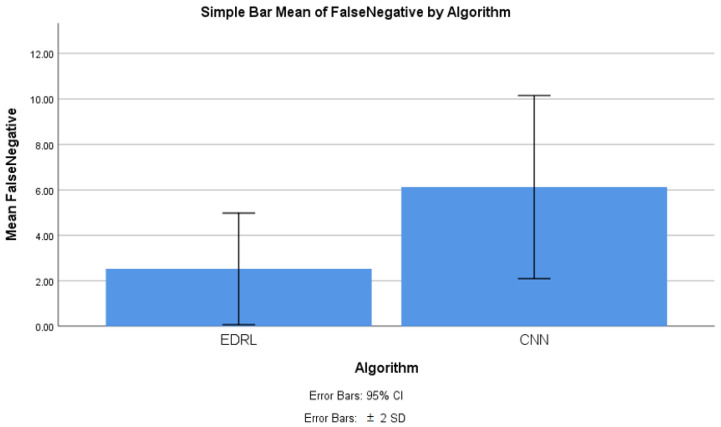
False negative comparison between EDRL and CNN algorithm with stand error bars with SD and confidence interval.

With the SPSS tool, the mean false negative of ERDL and CNN algorithms has been compared, as shown in Figure 6. The graph was generated with the SPSS graph builder, with the X axis as the ERDL vs. CNN algorithms, and the Y axis as the mean false negative. The standard deviation was set at ±2, and the confidence interval was 95%. The mean precision of the ERDL algorithm was higher than the CNN algorithm. For 10 epochs, the mean false negative was considerably higher for the ERDL than the CNN algorithm, and as the sample size increased, and the error rates decreased with increasing sample sizes for the two algorithms.

## 5. Discussion of Work

From the conducted experiment, the observation was made to infer the mean accuracy, mean precision, mean false positive, and mean false negative for the proposed EDRL algorithm; additionally, these parameters were compared with the CNN algorithm, in order to determine the performance measures of the entire algorithms, while considering the dataset with the data recorded at 54,000. From Figure 3, Figure 4, Figure 5 and Figure 6, it can be observed that the proposed EDRL algorithm outperformed on mean accuracy, mean precision, mean false positive, and mean false negative, when compared to the CNN algorithms performance measures. Initially the dataset contained 54,000. The dataset was collected from ISCXVPN2016 [20] for VPNs and non-VPNs. The size of the dataset is almost 15 GB of ARFF file format, with a set of attributes with instance sharing. A total of 80% of data were taken for training purposes, and 20% data were used for testing purposes.

The EDRL algorithm consumed less storage and computation time than the CNN algorithms, and this reduced consumption is an enhancement of the deep reinforcement learning algorithm EDRL. The network traffic prediction was achieved with a mean accuracy of 97.20% using the EDRL algorithm, which is higher than the CNN algorithm’s mean accuracy of 93.055%. Further, for the network traffic prediction, mean precision was achieved at 97.343% and 93.972%, respectively, for the EDRL and CNN algorithms. The mean false positive measures was achieved at 2.657% and 6.325%, respectively, for the EDRL and CNN algorithms. Finally, the mean false negative was achieved at 2.527% and 5.675%, respectively, for EDRL and CNN algorithms. The confusion matrix of algorithms were plotted to predict the traffic prediction and compared with the trained and testing dataset. When the numbers of iterations were increased, there was linear growth in the accuracy, precision, false positive, and false negative for the EDRL, which were better than that seen with the CNN algorithm. For the standard deviation, the standard error was tabulated for the algorithms. The significant difference was slightly better for the ERDL algorithm than the CNN algorithm. Finally, the performance measuring parameters were also measured with different samples of the dataset for EDRL and CNN, and it was observed that EDRL performed better than the CNN algorithms, in regard to mean accuracy, mean precision, mean false positive, and mean false negative.

The previous studies were measuring prediction accuracy using the KNN and RF [13,20,21,22], and the accuracy was achieved at 72.08% and 90.53%, respectively, with EDONKEY application network traffic. The artificial neural network [23] was used to measure the network traffic, with performance measures regarding the capacity of the network and traffic loss. Further, other studies claimed equivalent accuracy or more or less for traffic prediction, based on the types of network traffic. The application-oriented network traffic achieved comparatively less accuracy than the EDRL for network traffic of Amazon using the ANN algorithm (95.00%) [20,21], compared to 80.00% for EDONKEY traffic and 78.00% for FTP_CONTROL. For the network traffic of FTP and P2P, accuracy and precision were achieved at 94% and 90%, respectively, for the KNN algorithm [13,22]. The CNN-based application identification task accuracy was achieved at 94.00% [23]; when compared to the EDRL algorithm, the accuracy of application traffic classification was less with the UNB ISCX VPN-nonVPN dataset. Another work used SVM for network traffic and classification. The accuracy measure was reached at 94.2% [24]. This work has tested the SVM’s versions, as well, in order to measure the accuracy and precision. A comparative performance measure of various ML and DL algorithms were measured for KNN, RF, neural network (NN), and naïve Bayes (NB) as 79.6%, 84.8%, 84.6%, and 87.6%, respectively. This work has used a real time dataset of the orange platform of Nigerian University [1,25]. Table 13 presents various ML- and DL-based algorithm’s accuracy comparisons with the proposed EDRL algorithm.

The novelty of this work is that the EDRL gained reward-based output, comparatively more than existing the ML and DL algorithms. The reward-based decision-making policy is gained from the EDRL algorithm. Here, the accuracy and precision, as well as false positive and negative rates, were comparatively high, as discussed in Section 4 and Figure 3, Figure 4, Figure 5 and Figure 6.

The factors affecting the network traffic predictions are round trip delay time, application type with dynamic nature, payload of network data, etc. The proposed algorithm EDRL maintains some limitations, if the above factors are to be incorporated. Further, in the future, it is essential to incorporate limitations when enhancing the proposed work for the automation of network traffic predictions, as well.

## 6. Conclusions and Future Works

This research was carried out to predict the fine-tuned accuracy and precision, as well as the false positive and negative rates, for the network traffic of various types and classes of networks, in order to fill the research gap regarding the lack of algorithms for measures. An EDRL algorithm was coined, in order to get best results for taking parameters. The conducted experiments’ results illustrate that the EDRL algorithm is best with mean accuracy, mean precision, mean false positive, and mean false negative in both the numerical and graphical results.

This work could be used in various applications, such as network traffic prediction applications related to surveillance, sensitive types of traffic predictions, and other commercial applications, in order to monitor application traffic. This work could be extended for the automation of network traffic prediction by introducing an extended algorithm of EDRL for real time network traffic of surveillance and FTP traffic. That would be best, in order to deal with real time network traffic in the future.

## Figures and Tables

**Table 1:** Various classes and methods of machine and deep learning techniques.

Class	Method	Learning Technique
LSTM	Discriminative	Supervised
CNN		
RNN		
MLP		

**Table 2:** Data input, policy relationship for types of ML and DL algorithms.

Method	Data Input	
	Know Answer	Policy/Problems
Supervised learning	Learned output with supervision	Learning reward-based output with supervision
Reinforcement learning method	Maximize reward-based output	Feedback trained maximized reward-based output
Deep learning method	Deep learning-based output	Deep learning and feedback trained-based output
Deep reinforcement learning method	Deep-based maximize reward-based output	Deep and feedback trained-based maximize reward-based output
Enhanced deep reinforcement learning method	Accurate deep-based maximize reward-based output	Accurate output, based on deep and feedback trained maximize reward

**Table 3:** Network data features and its notation.

Feature Index	Notation	Feature Description
f_1_	avg_seg_sz	Average size of segment
f_2_	win_sz	Window size
f_3_	r_t_t	Round Trip delay Time
f_4_	var_pack	Variance in packets
f_5_	Act_dt_pkt	Actual data packet
f_6_	clt_pn	Client port number
f_7_	svr_pn	Server port number

**Table 4:** Classes of network traffic.

Class Index	Notation	Class Description	Applications
c_1_	www_pkt	www packet	General browsing data
c_2_	p2p_pkt	P2P network packet	Torrent streaming
c_3_	ml_pkt	Mail service packet	SMTP, POP, MIME, IMAP
c_4_	db_pkt	Database packet	SQL net
c_5_	mul_pkt	Multimedia packet	Video storage server YouTube

**Table 5:** Group statistics for accuracy comparison of EDRL vs. CNN algorithms to measure mean, standard deviation, and standard error mean.

Group Statistics					
	Algorithm	N	Mean	Std. Deviation	Std. Error Mean
Accuracy	EDRL	10	97.200	1.71156	0.538
	CNN	10	93.055	2.29835	0.727

**Table 6:** Comparison of the independent sample *t*-test parameters of EDRL and CNN algorithms.

Independent Samples Test										
		Levene’s Test for Equality of Variances		*t*-Test for Equality of Means						
		F	Sig.	t	df	Sig. (2-Tailed)	Mean Difference	Std. Error Difference	95% Confidence Interval of the Difference	
									Lower	Upper
Accuracy	Equal variances assumed	1.111	0.306	4.519	18	0.000	4.095	0.90619	2.191	5.999
	Equal variances not assumed			4.519	16.634	0.000	4.095	0.90619	2.180	6.010

**Table 7:** Group statistics for precision comparison of EDRL vs. CNN algorithms to measure mean, standard deviation, and standard error mean.

Group Statistics					
	Algorithm	N	Mean	Std. Deviation	Std. Error Mean
Precision	EDRL	10	97.343	1.519	0.480
	CNN	10	93.972	2.403	0.760

**Table 8:** Comparison of independent samples *t*-test parameters of EDRL and CNN algorithms for precision.

Independent Samples Test										
		Levene’s Test for Equality of Variances		*t*-Test for Equality of Means						
		F	Sig.	t	df	Sig. (2-Tailed)	Mean Difference	Std. Error Difference	95% Confidence Interval of the Difference	
									Lower	Upper
Precision	Equal variances assumed	2.351	0.143	4.295	18	0.000	3.861	0.899	1.972	5.750
	Equal variances not assumed			4.295	15.20	0.001	3.861	0.899	1.947	5.780

**Table 9:** Group statistics for false positive comparison of EDRL vs. CNN algorithms to measure the mean, standard deviation, and standard error mean.

Group Statistics					
	Algorithm	N	Mean	Std. Deviation	Std. Error Mean
False positive	EDRL	10	2.657	1.85335	0.586
	CNN	10	6.325	2.19063	0.693

**Table 10:** Comparison of independent samples *t*-test parameters of EDRL and CNN algorithms for false positive.

Independent Samples Test										
		Levene’s Test for Equality of Variances		*t*-Test for Equality of Means						
		F	Sig.	t	df	Sig. (2-Tailed)	Mean Difference	Std. Error Difference	95% Confidence Interval of the Difference	
									Lower	Upper
False positive	Equal variances assumed	0.372	0.550	−4.042	18	0.001	−3.668	0.907	−5.574	−1.762
	Equal variances not assumed			−4.042	17.51	0.001	−3.668	0.907	−5.578	−1.758

**Table 11:** Group statistics for false negative comparison of EDRL vs. CNN algorithms to measure the mean, standard deviation, and standard error mean.

Group Statistics					
	Algorithm	N	Mean	Std. Deviation	Std. Error Mean
False negative	EDRL	10	2.5270	1.22734	0.38812
	CNN	10	5.6750	1.9920	0.61643

**Table 12:** Comparison of independent sample *t*-test parameters of the EDRL and CNN algorithms for the false negative.

Independent Samples Test										
		Levene’s Test for Equality of Variances		*t*-Test for Equality of Means						
		F	Sig.	t	df	Sig. (2-Tailed)	Mean Difference	Std. Error Difference	95% Confidence Interval of the Difference	
									Lower	Upper
False negative	Equal variances assumed	3.113	0.095	−4.826	18	0.00	−3.598	0.746	−5.164	−2.032
	Equal variances not assumed			−4.826	14.87	0.00	−3.598	0.746	−5.188	−2.008

**Table 13:** Various ML and DL accuracy measure comparisons with the proposed EDRL algorithm.

Work Name	Algorithm Used	Accuracy
EDONKEY application network traffic [20]	KNN and RF	72.08% and 90.53%
FTP_CONTROL [20,22]	ANN	78.00%
The network traffic of FTP and P2P [13,23]	KNN	94%
The CNN based application identification task [21]	CNN	94%
Traffic classification was less with UNB ISCX VPN-Non-VPN dataset [24]	SVM	94.2%
Orange platform of Nigerian University [1]	KNN, RF, NN, and NB	79.6%, 84.8%, 84.6%, and 87.6%
Internet traffic of different applications	The proposed EDRL algorithm	97.20%

## Data Availability

Not applicable.
